# Sperm Selection Using Microfluidic Techniques Significantly Decreases Sperm DNA Fragmentation (SDF), Enhancing Reproductive Outcomes: A Systematic Review and Meta-Analysis

**DOI:** 10.3390/biology14070792

**Published:** 2025-06-30

**Authors:** Alma Gisbert Iranzo, Marina Cano-Extremera, Irene Hervás, Mar Falquet Guillem, María Gil Juliá, Ana Navarro-Gomezlechon, Rosa María Pacheco-Rendón, Nicolás Garrido

**Affiliations:** IVIRMA Global Research Alliance, IVI Foundation, Instituto de Investigación Sanitaria La Fe (IIS La Fe), Avenida Fernando Abril Martorell, 106—Torre A, Planta 1ª, 46026 Valencia, Spain; almagisbert99@gmail.com (A.G.I.); marina.cano99@gmail.com (M.C.-E.); irene.hervas@ivirma.com (I.H.); marfalquetuv@gmail.com (M.F.G.); mariagil.email@gmail.com (M.G.J.); ana.navarro@ivirma.com (A.N.-G.); rosa.pacheco@ivirma.com (R.M.P.-R.)

**Keywords:** microfluidics, male infertility, ICSI, in vitro fertilization, reproduction

## Abstract

This study aimed to compare sperm parameters and reproductive outcomes after sperm selection using microfluidic chips versus conventional techniques (swim-up/density gradients). A systematic review and meta-analysis were performed after the extraction of relevant data from thirty-nine studies that met the inclusion criteria. Mean difference or odds ratio was calculated for each outcome. The analysis revealed that sperm selection using microfluidics yields lower sperm DNA fragmentation (MD = −9.98 [−13.19, −6.76], *p* < 0.00001), increased progressive motility (MD = 14.50 [7.84, 21.71], *p* = 0.04), total motility (MD = 10.68 [6.04, 15.31], *p* < 0.00001) and morphology (MD = 1.41 [0.67, 2.16], *p* = 0.0002). Significant differences were also found in the fertilization rate/MII oocyte microinjected (OR = 1.22 [1.01, 1.46], *p* = 0.04), implantation rate/embryo transfer (ET) (OR = 4.51 [1.42, 14.37], *p* = 0.01), clinical pregnancy/ET (OR = 1.73 [1.22, 2.45], *p* = 0.002), ongoing pregnancy/ET (OR = 1.99 [1.03, 3.83], *p* = 0.04), live birth rate/first cycle (OR = 1.59 [1.12, 2.24], *p* = 0.009) and per all embryo transfer (OR = 1.65 [1.06, 2.55], *p* = 0.03). No significant differences were found in embryo euploidy/number of biopsied blastocysts (OR = 1.34 [0.88, 2.04], *p* = 0.77), biochemical pregnancy/ET (OR = 1.23 [0.84, 1.80], *p* = 0.29), miscarriage rate/cycle (OR = 0.84 [0.54, 1.31], *p* = 0.35) and per pregnancy (OR = 0.71 [0.50, 1.02], *p* = 0.07), live birth rate/first embryo transfer (OR = 1.60 [0.80, 3.22], *p* = 0.18) and per concluded cycle (OR = 1.03 [0.53, 2.00], *p* = 0.92). To summarize, microfluidics may offer a beneficial approach in certain situations, particularly for patients with elevated sperm DNA fragmentation (SDF) levels. However, its integration into routine clinical practice cannot be justified yet in terms of cost-effectiveness. Additional research is needed to provide more comprehensive data on reproductive outcomes, especially live birth rates, which remain the ultimate goal of assisted reproductive technologies.

## Simple Summary

Despite the remarkable technological advances and increasing success rates in the last decades, assisted reproduction techniques’ success is limited, frequently needing multiple treatments before achieving healthy offspring. Sperm selection is one of the key aspects to be improved, since single sperm are genetically unique, and its proper selection could enhance success rates in IVF/ICSI treatments. Microfluidic techniques have emerged in recent years as a promising tool that could revolutionize conventional sperm selection (through swim-up and/or density gradient techniques). Our results indicate that its use enables better sperm parameters, highlighting lower sperm DNA fragmentation (SDF), and improves some reproductive outcomes in intracytoplasmic sperm injection cycles. However, careful interpretation of these results is advised due to the variability in study populations and inconsistencies in the quality of some studies included in the analysis.

## 1. Introduction

Currently, around 15% of couples of reproductive age face infertility, defined as the inability to conceive after one year of unprotected sexual intercourse. This condition may arise due to female, male or combined known factors with a remaining relevant proportion of idiopathic infertility, where no apparent cause can be identified. In some cases, these factors directly cause infertility, while, in others, may diminish the chances of reproductive success [1,2].

In response to the aforementioned situation, assisted reproductive techniques (ARTs), following a proper diagnosis of infertility, aim to increase the chances of achieving a live birth (LB). They do so by replicating the physiological processes that naturally occur in vivo within the female reproductive tract, overcoming infertility factors and increasing the likelihood of reproductive success. However, these techniques’ success rates do not reach their full potential, frequently requiring repeated treatments due to diverse reasons (ovarian stimulation failures, poor embryonic development, implantation failure, recurrent miscarriages, etc.). As such, each step is in continuous development and improvement [3].

ART procedures require semen sample preparation to select the most competent sperm, focusing on optimal motility and morphology, which typically involve conventional techniques such as swim-up (SU) and density gradients (DGs). These techniques have shown to be safe and effective, primarily selecting sperm based on motility [3]. However, they do not consider the sperm’s underlying physiology or molecular profile. Both techniques involve sample manipulation, including pipetting and multiple centrifugation steps, which may negatively affect sperm viability by promoting sperm DNA fragmentation (SDF). This is crucial because, just as poor motility hinders sperm’s ability to reach the oocyte, sperm’s DNA damage has been associated with poorer embryonic quality and development, as well as an increased risk of miscarriage [1,4,5].

**Figure A1:**
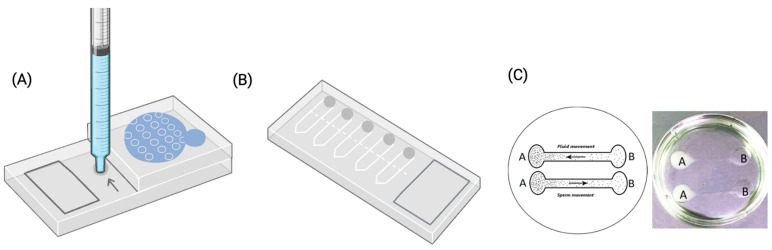
Graphical representation of the three groups of microfluidic chips used in the publications included for analysis. (**A**) ZyMot group. The arrow indicates where the semen sample is loaded into de device. (**B**) Fertile group by Koek Biotechnology. In this case the sample is introduced through the shaded regions. (**C**) ‘Others’ group, exemplified by the lab-made chip developed by Anbari et al. 2021 [20]. It is important to note that each author in this group designed their own chip, which differs in configuration and characteristics. While some studies provided detailed chip designs, others did not disclose specific structural information.

As a result, there has been a growing effort in recent years to develop new sperm selection methods that, in addition to being safe, replicate the physiological process of the female tract more efficiently. These techniques aim to minimize sample manipulation and consequent SDF while selecting sperm based on morphological and molecular features. Such is the use of testicular sperm [6], Magnetic Activated Cell Sorting (MACS) [7] or microfluidic chips. Testicular sperm has proven to be effective due to its retrieval prior to exposure to the oxidative environment of the seminiferous tubules and epididymis, where excessive production of reactive oxygen species (ROS) can induce oxidative stress and lead to SDF [6]. In contrast, techniques such as MACS and microfluidics are based on the selection of spermatozoa after ejaculation. Regarding microfluidic devices, they aim to mimic natural sperm selection by using microchannels, eliminating centrifugation steps and minimizing sample manipulation. Various devices are available, including the widely used ZyMot Multi, ZyMot ICSI (DxNow Inc., Gaithersburg, MD, USA) and Fertile Plus chip (Koek Biotechnology, İzmir, Turkey), while some authors have designed their own laboratory devices [4,8]. The difference between these two commonly used microfluidic chips (ZyMot and Fertile Plus) lies in their internal architecture. ZyMot utilizes a system of parallel microchannels without physical barriers or membranes, requiring spermatozoa to actively swim through narrow pathways from the inlet to the outlet chamber. In contrast, Fertile Plus consists of two chambers separated by a microporous membrane. Sperm selection occurs through passive filtration, where only spermatozoa with adequate morphology and appropriate size can traverse the membrane into the collection chamber [9,10,11]. An illustrative representation of each type of chip is provided in Appendix A (Figure A1).

In addition to the possible benefits from the use of microfluidic chips in relation to the elimination of the centrifugation steps, their use could also facilitate the workflow and allow freeing up technicians for other tasks. However, their implementation represents an additional cost for both the laboratory and patients undergoing infertility treatments. Additionally, while some studies have shown that microfluidics may improve sperm parameters and reproductive outcomes [4,5,10,12,13], other authors have reported no significant differences when compared to conventional techniques [1,14,15,16]. This highlights the lack of consensus, raising questions about the true potential benefits of its integration into daily clinical practice, although the reasons behind these discrepancies may include differences in the patient populations studied, protocols, outcomes measured, sample size and a number of other methodological factors leading to varying results in each study.

Despite the lack of consensus and robustness, the use of these systems in laboratories (currently categorized as add-ons due to the above-mentioned reasons) has increased worldwide over the years, being frequently offered to patients with different infertility etiologies [9]. Hence, there is a need to systematically analyze all available data to determine if incorporating this technique into routine clinical practice provides a proper cost–benefit balance. The aim of this systematic review and metanalysis is to evaluate whether sperm parameters, as well as reproductive outcomes in assisted reproduction cycles, are improved, and by how much, based on the sperm selection technique using either conventional techniques (SU/DG) or microfluidic devices.

## 2. Materials and Methods

This meta-analysis was conducted following the PRISMA (Preferred Reporting Items for Systematic Reviews and Meta-Analyses) 2020 guidelines [17] and registered in the PROSPERO database (ID: CRD42024582267). This registration not only ensures the transparency and accuracy of the research process but also allows other researchers to access information about the methods and objectives of our study.

### 2.1. Search Strategy

An automated search was conducted in the PubMed and Scopus databases, along with a manual reference search of the identified studies, according to our protocol and following the PRISMA guidelines. The same search strategy was used in each case: sperm AND microfluidic AND human AND (“reproductive results” OR “DNA fragmentation”). All (human) study types were considered, except for systematic reviews or meta-analyses. These searches were conducted between October 2023 and October 2024.

### 2.2. Study Identification and Selection

Using the described search strategy, a total of 196 studies were retrieved (158 from PubMed and 38 from Scopus), excluding literature reviews. Of these, 115 publications were excluded based on title or abstract. Of the remaining 81, 21 were removed as duplicates. After full-text review, 39 studies (articles, abstracts and posters from conferences) were included, while the final exclusions were due to ambiguous or non-useful results for statistical analysis, failure to compare with conventional sperm selection techniques (SU/DG) or full text unavailability. From the 39 publications, 9 were prospective randomized studies and 30 non-randomized (with 16 prospective and 14 retrospective studies).

The studies that were included were those in which (i) the authors performed a comparation between seminal parameters after semen samples (from male patients, volunteers or under fertility treatments) were processed either using microfluidics or a conventional technique (SU/DG); (ii) the authors performed a comparation between seminal parameters and/or reproductive outcomes from patients undergoing AI/IVF/ICSI cycles after sperm selection using either microfluidics or a conventional technique. Studies that did not present a comparison, had no control group (SU/DG), there was important missing data or presented ambiguous or non-useful data for the statistical analysis were excluded from the selection.

### 2.3. Data Extraction and Database Creation

Relevant data were extracted and entered into an Excel database, in which the information was structured for statistical analysis. Data included both semen parameters (SDF (%), concentration (M/mL), motility (%) and morphology (%)) and reproductive outcomes (%) from ART (fertilization rates/metaphase II (MII) oocyte microinjected, euploidy/number of biopsied blastocysts, implantation/embryo transfer (ET), pregnancy/ET, miscarriage per cycle and per pregnancy, live birth rate (LBR) per 1st ET, 1st cycle, all ET and concluded cycle). Article inclusion was reviewed by two researchers (A.G.I. and M.C.), with a third (N.G) as a tiebreaker in case of disagreement. All authors have read and agreed to the published version of the manuscript.

### 2.4. Statistical Analysis

The statistical analysis was conducted using Review Manager 5, developed by the Cochrane Collaboration [15]. Data from the selected studies were recorded and classified by either seminal parameters or reproductive outcomes. Control (SU and/or DG) and experimental procedures (microfluidics) were compared to obtain odds ratios (ORs) or mean differences (MDs), as appropriate, along with the corresponding 95% confidence intervals (CIs). Continuous variables (such as seminal parameter measurements) were grouped using the inverse of the variance model, with the results represented as mean differences. For non-continuous variables, the results were presented as ORs. The Mantel–Haenszel model was applied considering differences significant at *p*-values < 0.05.

To assess heterogeneity and variations between studies not attributable to sampling errors, the I^2^ statistic was used. Values above 50% were considered indicative of substantial heterogeneity. Due to the inherent variability found across the studies, only random effects were considered, regardless of the I^2^ values found. Homogeneity was generally low, as determined by the different research designs and methodologies across the studies evaluated, and also as indicated by I^2^, which were almost always above 50%. For this reason, random effects models were preferred when conducting the statistical analysis.

Funnel plots were generated to explore potential publication bias in the studies through a qualitative assessment of their symmetry. Consequently, forest plots to assess subgroup analyses were performed (microfluidics chip type: ZyMot, ZyMot Fertility, MD USA; Fertile Plus, Koek Biotechnology, Turkey; Other, custom devices by authors and other commercial brands) for each seminal or reproductive outcome to identify potential sources of heterogeneity.

### 2.5. Evaluation of the Methodological Quality

**Figure A2:**
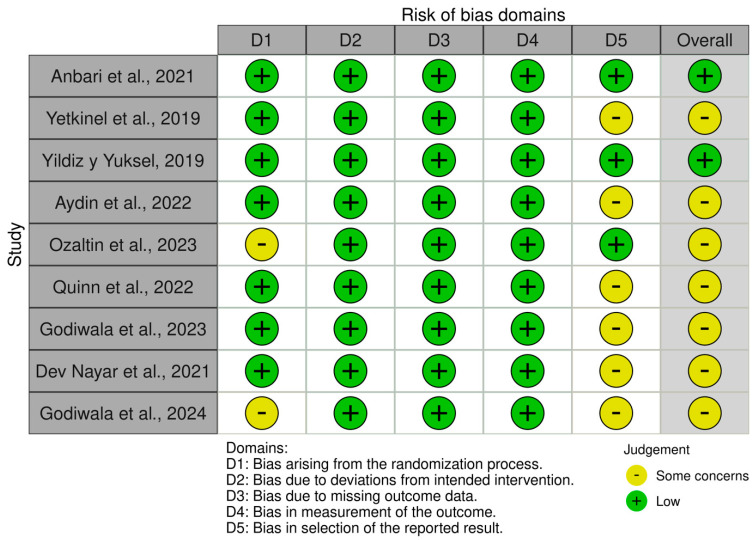
Representation of the quality analysis based on the presence of risk of bias in the randomized studies [1,14,20,24,29,34,40,41,43] included in the meta-analysis, using the RoB 2 tool. The legend displays the color code used, as well as the definition of each domain for which the presence/absence of bias was determined.

**Figure A3:**
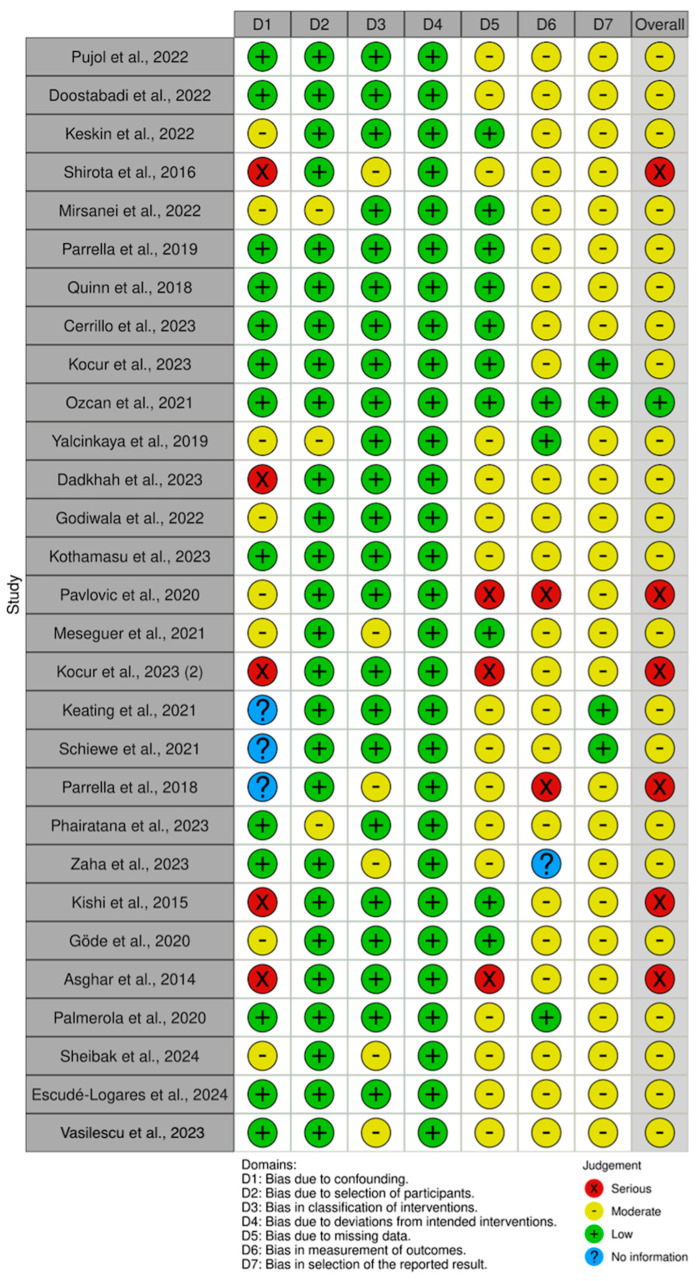
Representation of the quality analysis based on the presence of risk of bias in the non-randomized studies [4,5,8,10,11,12,14,16,21,23,25,26,27,28,30,31,32,33,35,36,37,38,39,42,44,45,46,47] included in the meta-analysis using the ROBINS-I tool. The legend presents the color code used, as well as the definition of each domain for which the presence/absence of bias was determined.

The quality in terms of risk of bias of the publications included in the meta-analysis was assessed using the RoB 2 [18] and ROBINS-I [19] tools. Each evaluation included multiple domains assessing specific aspects of bias, allowing the determination of both individual domain risk and the overall risk of bias for each publication. The RoB 2 tool was applied when publications were randomized trials, while ROBINS-I was used for non-randomized studies. The results of the quality assessment are presented in Figure A2 and Figure A3, respectively.

## 3. Results

**Figure 1:**
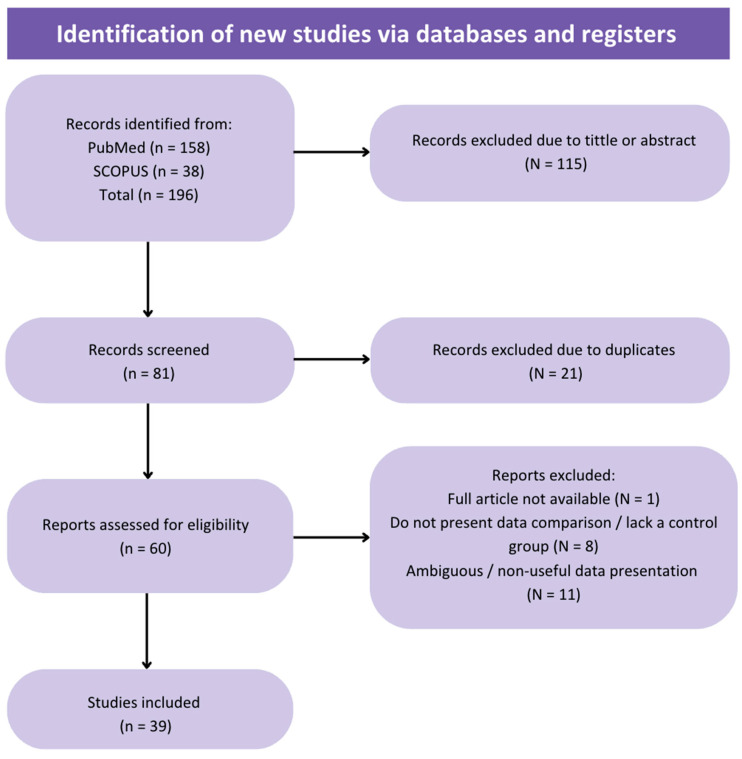
Flow diagram of the review process and selection of studies included in the meta-analysis.

As previously mentioned, a total of 39 studies were included in the meta-analysis, and the selection process is shown in Figure 1 (Flow diagram). From the 39 studies, 9 were randomized controlled trials (RCTs) and 30 non-randomized. For each analyzed parameter, all studies presenting data were considered, regardless of whether they were RCTs or non-randomized studies. The main characteristics of the included studies, such as the design, study population, type of microfluidic chip used and measured parameters, are summarized in Table A1.

It is recommended to consider the quality analysis when evaluating the following results. Risk of bias was assessed as low (high quality) in two randomized trials (Figure A2), while seven articles were assessed as having a moderate risk of bias (moderate quality). For non-randomized trials, the quality results exhibited greater variability (Figure A3), with 1 study rated as low risk of bias, 23 publications with moderate risk of bias, and 6 articles rated as serious risk of bias (low quality).

**Figure A4:**
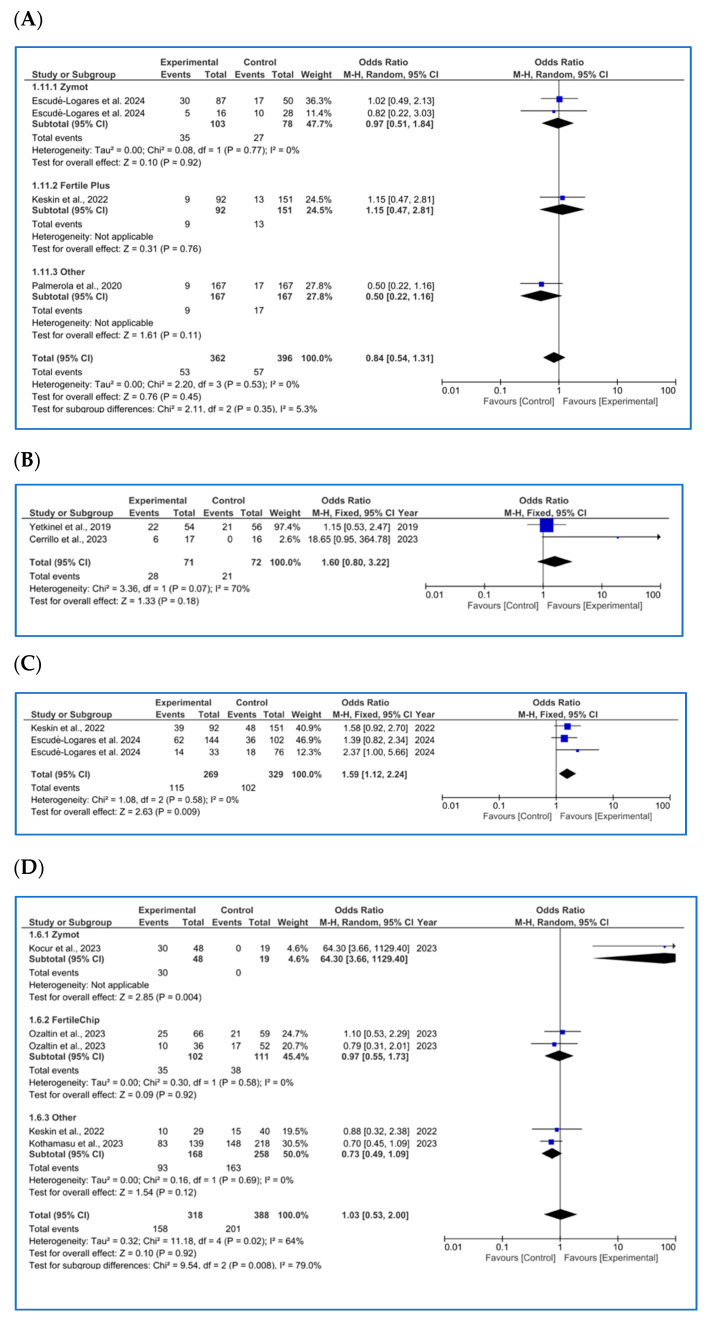
Forest plot with odds ratio (OR) values for studies that provide data on reproductive outcomes comparing the use of microfluidic chips with conventional sperm selection techniques (SU/DG). An OR > 1 indicates a significant difference in favor of the use of microfluidics and vice versa. (**A**) Miscarriage rate per cycle [26,35,44]. (**B**) LBR per first transfer [18,45]. (**C**) LBR per first cycle [35,44]. (**D**) LBR per completed cycle [8,35,36,41].

Additionally, funnel plots were used to analyze the heterogeneity between the studies included in each seminal parameter/reproductive outcome (Figure A3 and Figure A4, respectively). These plots also enabled the assessment of publication bias based on the dispersion of points in the graph. In general, most graphs did not show symmetry in the points, with greater dispersion indicating the presence of publication bias, except for three parameters, with the funnels plots acquiring a pyramidal structure (less data dispersion and absence of publication bias): miscarriage rate per pregnancy (Figure A6H), LBR per first transfer (Figure A6I) and LBR per first cycle (Figure A6J). However, for the last two outcomes, the results are not entirely conclusive due to the limited sample size, as only three articles were included.

### 3.1. Seminal Parameters

**Figure 2:**
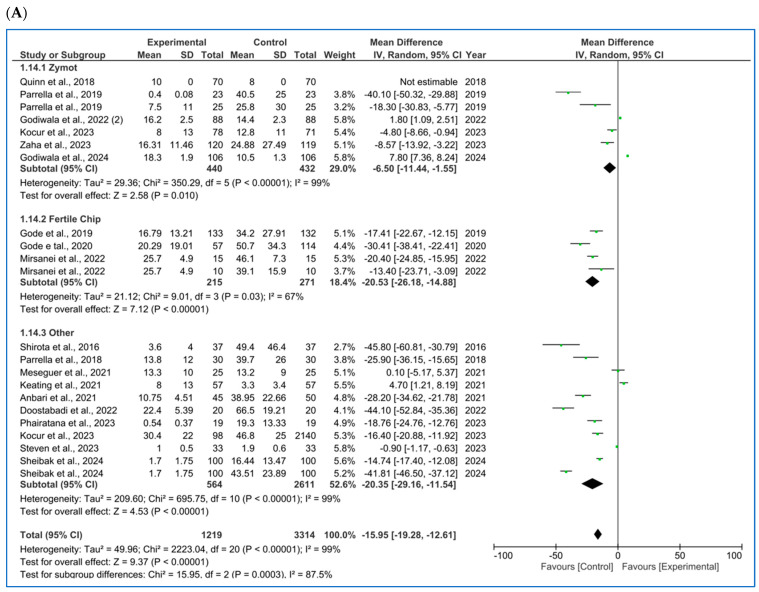

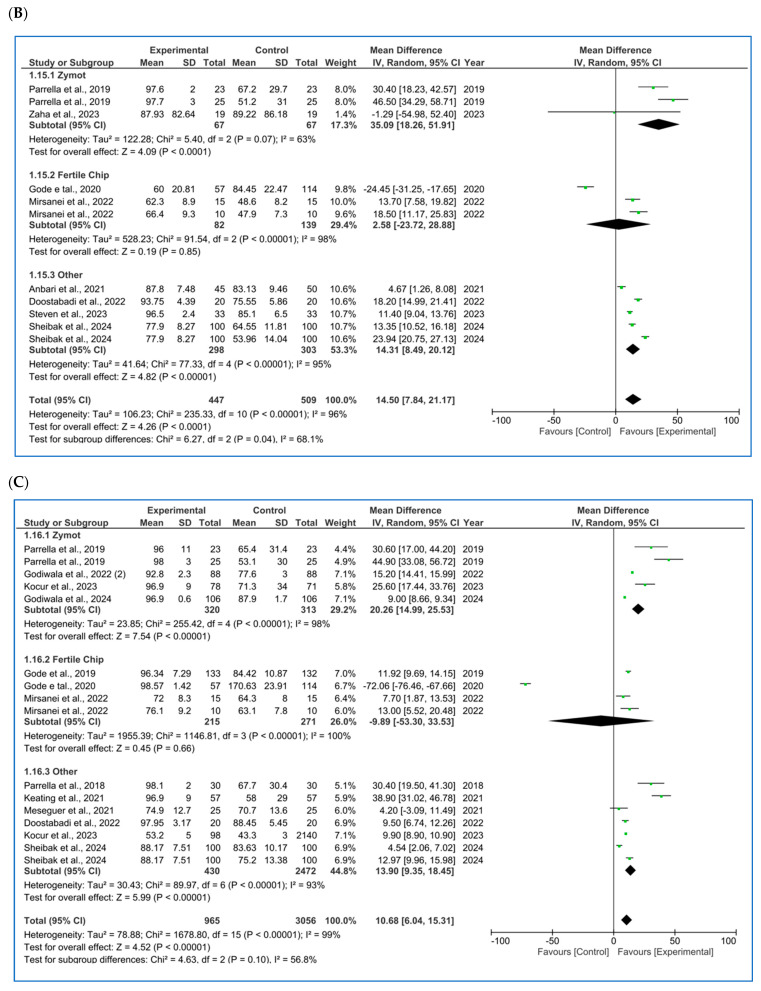

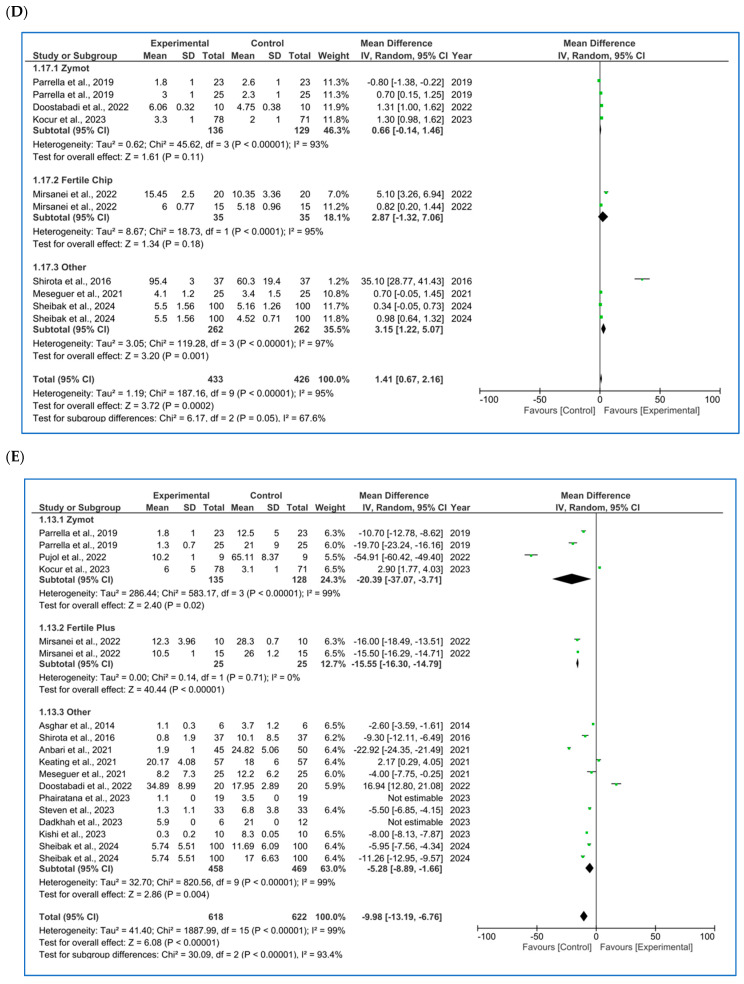
Forest plot displaying the mean differences (MDs) for studies providing data on seminal parameters, comparing the use of microfluidic chips with conventional sperm selection techniques (SU/DG). A positive MD (>0) indicates a significant difference in favor of the use of microfluidics and vice versa. (**A**) Results for concentration [4,10,11,16,20,21,22,23,24,25,26,27]. (**B**) Results for progressive motility [10,11,16,20,21,22,27]. (**C**) Results for total motility [4,10,11,21,22,23,25,27,28,29,30]. (**D**) Results for sperm morphology [10,11,21,25,27,30,31]. (**E**) Results for sperm DNA fragmentation (SDF) [4,5,10,11,12,20,21,25,27,30,31,32].

Starting with the analysis of the different seminal parameters, the following subsections detail the results obtained from the comparison between the two sperm processing techniques. These findings are summarized in Figure 2.

#### 3.1.1. Concentration

**Figure A5:**
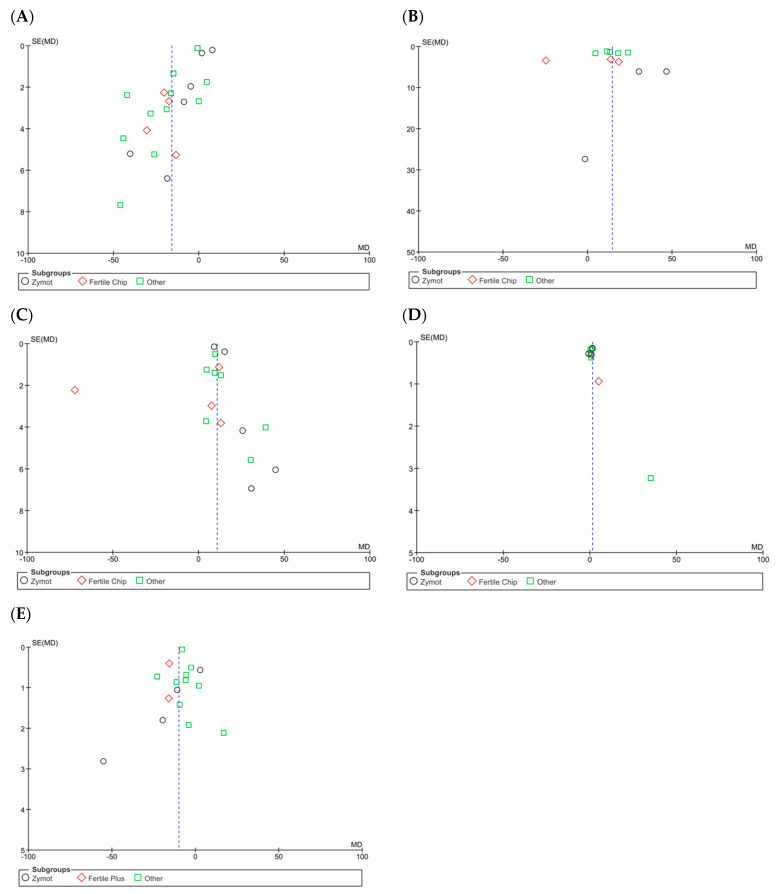
Funnel plots of the studies included in the analysis of the effect of microfluidic chip use on sperm parameters compared to conventional sperm selection techniques (SU/DG). Its visual interpretation determines the possible presence or absence of publication bias, based on whether it takes on a pyramidal structure or not, respectively. Dashed lines indicate the expected 95% confidence region, serving as a visual aid to assess the symmetry of the plot. (**A**) Results of concentration. (**B**) Progressive motility results. (**C**) Total motility results. (**D**) Sperm morphology results. (**E**) Sperm DNA fragmentation (SDF).

For sperm concentration (M/mL) [4,10,11,16,20,21,22,23,24,25,26,27] (Figure 2A), microfluids significantly lowered the concentration for each subgroup (ZyMot: *p* = 0.01, MD = −6.50 [−11.44, −1.55]; FertileChip: *p* < 0.00001, MD = −20.53 [−26.18, −14.88]; Others: *p* < 0.00001, MD = −20.3 [−29.16, −11.54]). The same conclusions were drawn in the combined analysis of all chip types (*p* < 0.00001, MD = −15.95 [−19.28, −12.61]). The heterogeneity was considerable (I^2^ = 99%, *p* < 0.00001), and the funnel plot (Figure A5A) suggests the possibility of publication bias.

#### 3.1.2. Motility

When analyzing progressive motility (%A+B) [10,11,16,20,21,22,27], microfluidics showed significant improvement of this parameter (Figure 2B) in the ZyMot (*p* < 0.0001, MD = 35.09 [18.26, 51.91]) and Other (*p* < 0.00001, MD = 14.31 [8.49, 20.12]) groups when compared to SU/DG. Within the ZyMot group, the two analyses conducted by Parrella et al. [11] led to this difference, as the study by Zaha et al. [33] crossed the line of no effect (though it had lower precision, with a wider 95% CI). No differences were found between FertileChip and the conventional techniques (*p* = 0.85, MD = 2.58 [−23.72, 28.88]). The forest plot when considering all chip types showed higher progressive motility when using microfluids (*p* = 0.04, MD = 14.50 [7.84, 21.17]). However, it should be noted that the heterogeneity in this case is also considerable, and the funnel plot (Figure A5B) suggests a potential publication bias.

As for the total motility (%A + B + C) [4,10,11,21,22,23,25,27,28,29,30] analysis (Figure 2C), FertileChip studies once more crossed the line of no effect (*p* = 0.66, MD = −9.89 [−53.30, 33.53]), while both ZyMot (*p* < 0.0001, MD = 20.26 [14.99, 25.53]) and Other (*p* < 0.00001, MD = 13.90 [9.35, 18.45]) showed a significant increase in total motility. When considering all chips, benefits for total motility were found with microfluidics (*p* < 0.00001, MD = 10.68 [6.04, 15.31]). Once again, the heterogeneity between studies was high, and publication bias seemed to appear in the funnel plot (Figure A5C).

#### 3.1.3. Morphology

Morphology (% normal forms) [10,11,21,25,27,30,31] was also analyzed (Figure 2D) following the aggregated approach, showing a beneficial effect when using microfluidics (*p* = 0.0002, MD = 1.41 [0.67, 2.16]). It is worth noting that the study by Parrella et al. [11], favored either conventional techniques or microfluidics depending on the study population (ICSI cycles vs. ICSI cycles after prior failures at other centers + PGT, respectively). This may help identify which cases could benefit most from its use. Again, considerable heterogeneity was detected (I^2^ = 95%) and publication bias was observed through the funnel plot (Figure A5D).

#### 3.1.4. Sperm DNA Fragmentation (SDF)

SDF had the substantially largest number of published studies [4,5,10,11,12,20,21,25,27,30,31,32], with a subsequent higher precision, as seen in its 95% CI in the forest plot (Figure 2E). SDF detection techniques have varied among authors, with TUNEL (terminal deoxynucleotidyl transferase dUTP nick end labeling) [4,11,25,30,32] and SCD (sperm chromatin dispersion) [10,20,21,27] being the most commonly used methods. However, some studies have also employed alternative techniques such as SCSA (sperm chromatin structure assay) [31], COMET (comet assay) [5] or Halosperm [12]. Since the subgroups of interest for the present analysis were based on the type of microfluidic chip used, the forest plot does not display a stratification by SDF detection method. Nevertheless, the specific methodology used in each study was duly recorded in the database.

Moreover, to enhance the trustworthiness of the analysis, we performed subgroup analysis based on both the type of microfluidic chip and the DNA fragmentation detection technique. However, no significant differences were found in either the *p*-values or the 95% confidence intervals when compared to the analysis presented in Figure 2E, in which we incorporated all the studies regardless of the SDF detection method used. Additionally, some methods, such as COMET or Halosperm, were reported in only one study, making subgroup analyses by each SDF detection method impractical due to the decrease in statistical power.

In the subgroup analysis, ZyMot chip showed significant differences (*p* = 0.02, MD = −20.39 [−37.07, −3.71]). All articles suggested the same findings, except for Kocur et al. [25], whose data determined that microfluidics did not improve the SDF, although it was very close to the line of no effect (*p* < 0.00001, MD = 2.90 [1.77, 4.03]). On the other hand, Mirsanei et al. [10] found significant differences favoring the use of FertileChip in two different analyses (*p* < 0.00001, MD = −15.55 [−16.30, −14.79]). In the Others group, there was greater variability among the studies, with some crossing the line of no effect, yet showing statistically significant improvement in the experimental group (MD −5.28 [−8.89, −1.66]).

In the overall analysis of all chip types, significant differences were obtained, demonstrating favorable results by lowering the SDF (*p* < 0.00001, MD = −9.98 [−13.19, −6.76]) when using microfluidics. Considerable heterogeneity was observed (I^2^ = 99%, *p* < 0.00001), and the funnel plot (Figure A5E) appears to suggest the presence of publication bias.

### 3.2. Artificial Insemination Outcomes (AI)

Only one retrospective study addressing the use of microfluidics in artificial insemination cycles was found [22]. Given that it was a single article, no statistical analysis was performed, but it is worth noting that better outcomes were reported when using FertileChip (compared to DG) in terms of biochemical pregnancy rate, clinical pregnancy and ongoing pregnancy, although none of the differences reached statistical significance (*p* > 0.05).

### 3.3. IVF-ICSI Reproductive Outcomes

All publications referred to ICSI cycles, with the exception of Palmerola et al.’s study [26], which was conducted in in vitro fertilization (IVF) cycles.

**Figure 3:**
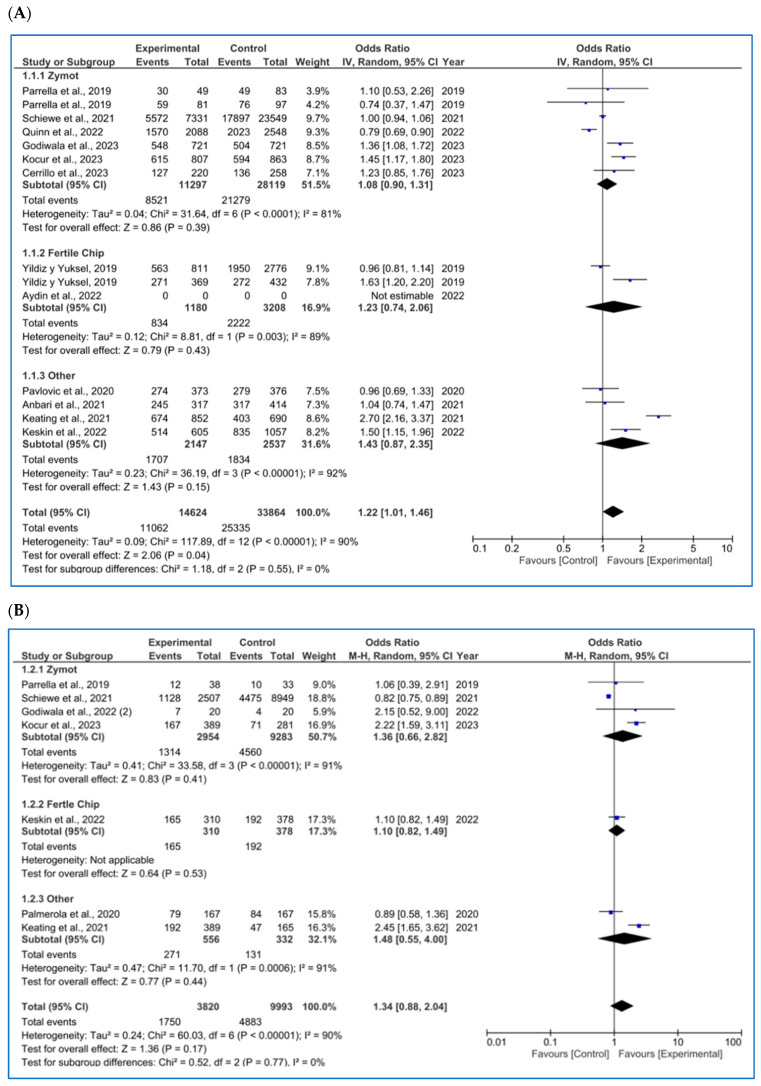

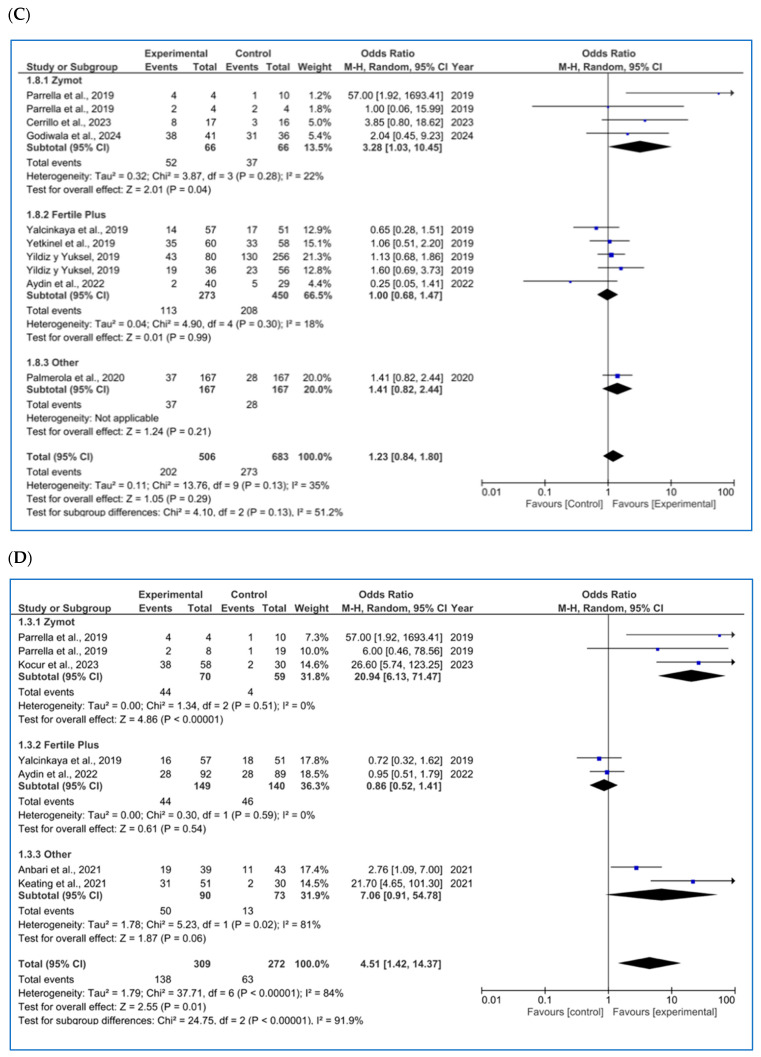

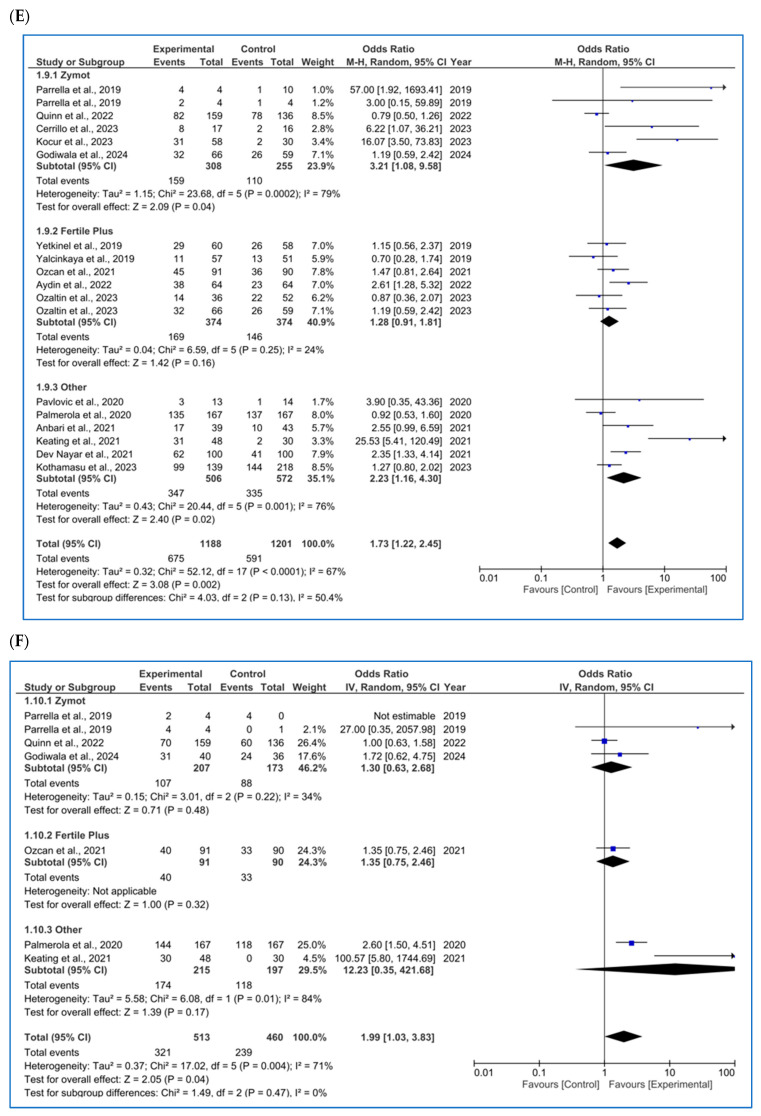

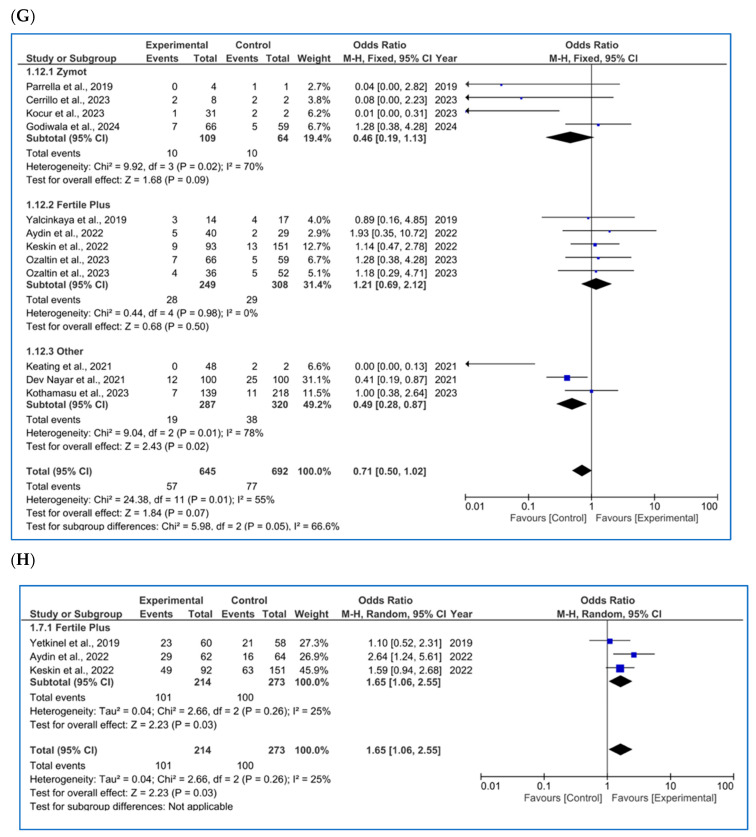
Forest plot displaying the odds ratios (OR) for studies providing data on reproductive outcomes, comparing the use of microfluidic chips with conventional sperm selection techniques (SU/DG). An OR > 1 indicates a significant difference in favor of the use of microfluidics and vice versa. (**A**) Fertilization rate [4,11,14,20,23,34,35,36,37,38,39]. (**B**) Embryo euploidy rate [4,11,24,26,35,36,38]. (**C**) Biochemical pregnancy rate [11,16,18,26,29,34,40]. (**D**) Implantation rate [4,11,16,20,34,36]. (**E**) Clinical pregnancy rate [1,4,8,11,14,16,18,20,26,29,34,36,37,41,42]. (**F**) Ongoing pregnancy rate [4,11,14,26,28,42]. (**G**) Miscarriage/pregnancy rate [1,4,8,11,16,28,34,35,36,41]. (**H**) Live birth rate/all embryo transfers [34,35,43].

Consistent with the previous section, the outcome-specific results are detailed in the following subsections. A comprehensive summary of these findings is also provided in Figure 3.

#### 3.3.1. Fertilization Rate

For the fertilization rate (per MII oocyte microinjected, %), as shown in Figure 3A, 12 studies were included [4,11,14,20,23,34,35,36,37,38,39]. No significant differences were found in any subgroup (ZyMot *p* = 0.39 OR = 1.08 [0.90, 1.31], FertileChip *p* = 0.43 OR = 1.23 [0.74, 2.06]; Others *p* = 0.15 OR = 1.43 [0.87, 2.35]). However, when considering the overall results, significant differences were found (*p* = 0.04), demonstrating an improvement in the fertilization rates when using microfluidics, although the OR (1.22 [1.01, 1.46]) presents a confidence interval close to the line of no effect.

**Figure A6:**
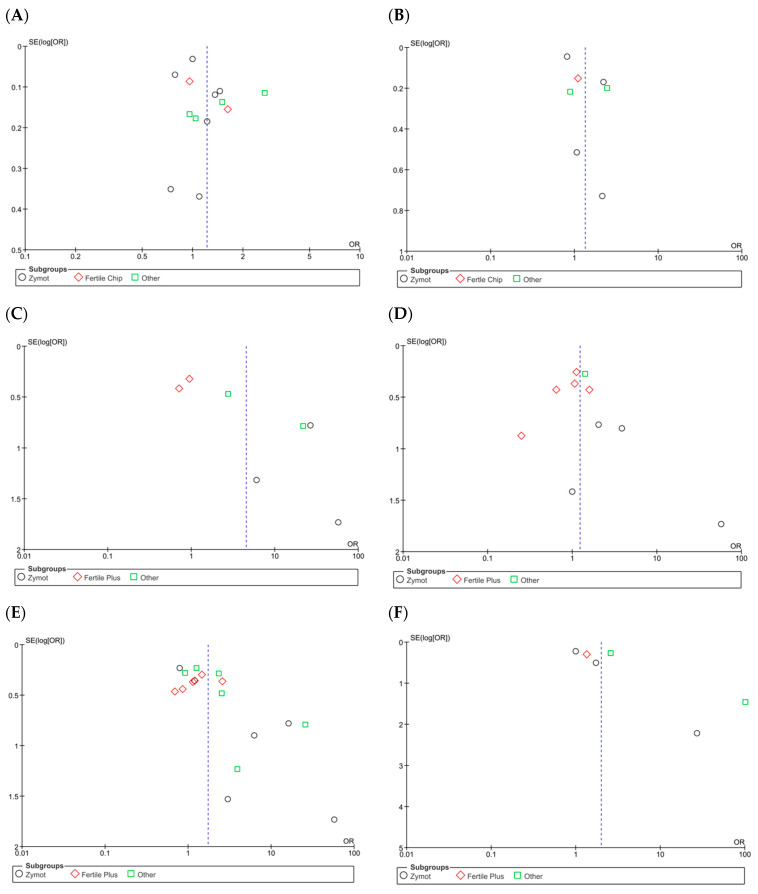

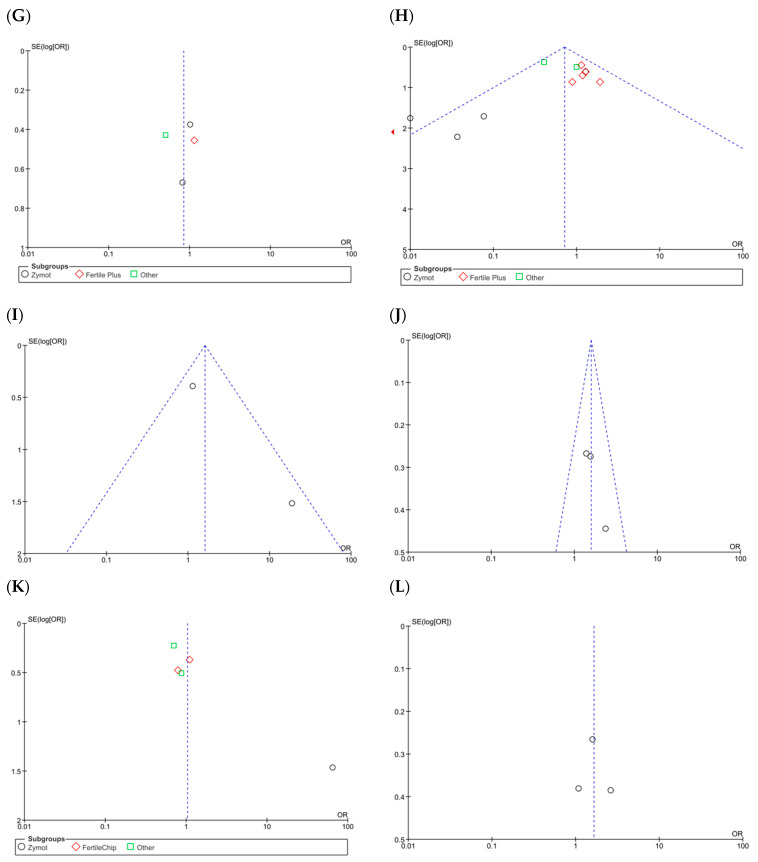
Funnel plots of the studies included in the analysis of the effect of microfluidic chip use on reproductive outcomes compared to conventional sperm selection techniques (SU/DG). Its visual interpretation determines the possible presence or absence of publication bias, based on whether it adopts a pyramidal structure or not, respectively. Dashed lines indicate the expected 95% confidence region, serving as a visual aid to assess the symmetry of the plot. (**A**) Fertilization rate results. (**B**) Euploid embryo rate. (**C**) Implantation rate. (**D**) Biochemical pregnancy rate. (**E**) Clinical pregnancy rate. (**F**) Ongoing pregnancy rate. (**G**) Miscarriage rate per cycle. (**H**) Miscarriage rate per pregnancy. (**I**) LBR per first transfer. (**J**) LBR per first cycle. (**K**) LBR per all embryo transfers. (**L**) LBR per completed cycle.

On the other hand, it is important to note the considerable heterogeneity among the studies (I^2^ = 90%, *p* < 0.00001). A dispersion of the articles is also observed in the funnel plot (Figure A6A), which does not form a pyramidal structure, indicating a potential publication bias.

#### 3.3.2. Embryo Euploidy Rate

For the rate of euploid embryos/number of biopsied blastocysts (%) (Figure 3B), seven studies were included in the analysis [4,11,24,26,35,36,38]. No significant differences were found between the two techniques (*p* = 0.77, OR = 1.34 [0.88, 2.04]), despite some authors reporting favorable results when using microfluidics [4,36]. The heterogeneity among the studies remained high (I^2^ = 90%, *p* < 0.00001). Once again, the funnel plot (Figure A6B) shows a potential publication bias.

#### 3.3.3. Biochemical Pregnancy Rate

For biochemical pregnancy rate/all ET (%), eight publications were found (Figure 3C) [11,16,18,26,29,34,40], and some of the results differed based on the study population [13,40]. No significant differences were found in the biochemical pregnancy rate based on the sperm selection protocol used by FertileChip (*p* = 0.99, OR = 1.00 [0.68, 1.47] and Others (*p* = 0.21, OR = 1.41 [0.82, 2.44]). Nevertheless, the ZyMot group showed improved biochemical pregnancy rates (*p* = 0.04, OR = 3.28 [1.03, 10.45]), with the 95% CI nearly crossing the line of no effect. In the overall microfluidic chip analysis, no significant differences were observed when compared to the conventional techniques (*p* = 0.29, OR = 1.23 [0.84, 1.80]), with the exception of Parella et al.’s study [11]; however, this study shows low precision with an OR of 57 [1.92, 1693.41]). Furthermore, we again encounter a non-pyramidal funnel plot, suggesting publication bias (Figure A6C).

#### 3.3.4. Implantation Rate

Regarding implantation rate/number of ET (%) (Figure 3D), six studies were included, with significant differences favoring the use of ZyMot [11,36] (*p* < 0.00001 (OR = 20.94 [6.13, 71.47])) and Other [4,20] (*p* = 0.06 (OR = 7.06 [0.91, 54.78])). However, it is worth mentioning that these studies had the greatest dispersion and therefore lower precision. Neither of the two studies [16,34] using FertileChip showed significant differences between techniques (*p* = 0.54, OR = 0.86 [0.52, 1.41]). When addressing the overall analysis, significant differences were demonstrated supporting the use of microfluidic devices (*p* = 0.01, OR = 4.51 [1.42, 14.37]). Considerable heterogeneity was detected (I^2^ = 84%, *p* < 0.00001), and the funnel plot indicates a potential publication bias (Figure A6D).

#### 3.3.5. Clinical Pregnancy Rate

Regarding clinical pregnancy rate/all ET (%), 16 articles were included [1,4,8,11,14,16,18,20,26,29,34,36,37,41,42] (Figure 3E), 2 [13,41] of them showing different results depending on the study population. In the FertileChip subgroup, no significant differences were found (*p* = 0.16, OR = 1.28 [0.91, 1.81]), unlike ZyMot (*p* = 0.04, OR = 3.21 [1.08, 9.58) or Other (*p* = 0.02, OR = 2.23 [1.16, 4.30]), where differences showed an improvement in the clinical pregnancy rate when using these chips. It is worth mentioning that studies on both ZyMot and Other showed high dispersion in their 95% CIs, an indicator of lower precision. In the overall analysis, significant differences were found, showing higher clinical pregnancy rates when using microfluidics (*p* = 0.002, OR = 1.73 [1.22, 2.45]).

Additionally, substantial heterogeneity between studies was observed (I^2^ = 67%, *p* < 0.0001). Although publications using Fertile Plus appear to be grouped considerably in the funnel plot (Figure A6E), those presenting results from ZyMot and custom-made chips are more dispersed, indicating a potential publication bias.

#### 3.3.6. Ongoing Pregnancy Rate

For ongoing pregnancy rate/all ET (%), with six articles included [4,11,14,26,28,42], significant differences (Figure 3F) were found when using microfluidics rather than the conventional methods. However, they were very close to the line of no effect (*p* = 0.04, OR = 1.99 [1.03, 3.83]). Additionally, some of the studies showed low precision due to data dispersion [4,13]. There was also substantial heterogeneity (I^2^ = 71%, *p* = 0.004), and the funnel plot did not show a pyramidal structure (Figure A6F).

#### 3.3.7. Miscarriage Rate (All Types)

Regarding miscarriage rate (biochemical and clinical, %), most of the studies included in the metanalysis were analyzed per pregnancy. Three of the publications did not clearly specify the denominator; therefore, it was inferred as per cycle based on the data presentation.

As mentioned, only three articles [26,35,44] were found per cycle (one using ZyMot, one FertileChip and one Other). The analysis showed no significant differences in the miscarriage rate per cycle (Figure A4A) based on the sperm selection method (*p* = 0.35, OR = 0.84 [0.54, 1.31]).

As for miscarriage rate per pregnancy (%) (Figure 3G), 11 studies were included [1,4,8,11,16,28,34,35,36,41]. Following the overall focused analysis, no differential effect was observed between techniques (*p* = 0.07, OR = 0.84 [0.54, 1.31]). The heterogeneity is moderate (I^2^ = 58%, *p* = 0.009), and the funnel plot (Figure A6H) shows a pyramidal structure, ruling out the presence of publication bias.

#### 3.3.8. Live Birth Rate

Based on the presentation of the data, this reproductive outcome was measured as LBR per first transfer, per first cycle, per all transfers or per completed cycle.

As for LBR per first transfer (%) (Figure A4B), only two studies [18,45] provided these results, with the first study showing a larger size effect. The overall analysis suggested that there were no significant differences in the clinical pregnancy rate per first transfer when using either microfluidic devices or conventional techniques, as the OR = 1.60 [0.80, 3.22] crossed the line of no effect (*p* = 0.18). The funnel plot shows a pyramidal structure, ruling out the presence of bias (Figure A6I).

For LBR per first cycle (%) (Figure A4C), only two publications were available [35,44], where Escudé-Logares et al. [44] provided data from two different study populations. An improvement in the LBR when using microfluidic devices (*p* = 0.009, OR = 1.59 [1.12, 2.24]) was found, although the size effect was small. In this case, the funnel plot shows a pyramidal structure. However, since only two articles are included, no conclusive statements can be made regarding publication bias (Figure A6J).

Regarding LBR per all embryo transfers (Figure 3H) [34,35,43], significant beneficial differences were found in microfluids (*p* = 0.03, OR = 1.65 [1.06, 2.55]). The heterogeneity in this section is substantial, I^2^ = 64 (*p* = 0.02), and the funnel plot indicates a potential publication bias (Figure A6K).

Lastly, four studies considered outcomes per concluded cycle (%) (Figure A4D). One study used ZyMot [36], one used FertileChip [41] and two used Other [8,35]. Except for Kocur et al. [36], the authors did not find significant differences between processing methods in terms of LBR rate per cycle. It is worth noting that Kocur et al.’s study had the lowest precision, with an OR = 64.30 [3.66, 1129.40] that contributed 4.6% to the overall size effect. Regarding the overall analysis, significant differences were found, suggesting an improvement in this rate when using microfluidics (*p* = 0.92, OR = 1.03 [0.53, 2.00]). The funnel plot does not present a pyramidal structure, which, in this case, may be due to the limited number of studies available for the analysis (Figure A6L).

### 3.4. Summary of the Analysis of the Seminal Parameters and Reproductive Outcomes in IVF/ICSI

A summary table (Table 1) provides an overview of the results obtained from the statistical analysis of each outcome. It includes the values for each outcome, the corresponding estimator with its 95% CI and the number of studies included in each analysis.

## 4. Discussion

This meta-analysis compiled all available studies evaluating the impact of microfluidic techniques on semen parameters and reproductive outcomes in ART, mainly focused on ICSI cycles. After selection, 39 studies were included for analysis.

Significant differences were found across all seminal parameters. Regarding sperm concentration, a significantly lower concentration was observed in the microfluidics group after processing. This effect is to be expected, as microfluidic devices allow a limited sample volume for processing, usually between 500 μL and 850 μL [9], whereas conventional methods can utilize up to the entire sample volume, accounting for the total sperm count available. This rationale was also noted by most authors [13,27]. In terms of sperm motility, analysis showed a significant improvement in both progressive and total motility when using microfluidics, according to the literature [4,23]. This suggests that, as sperm migrate through the microfluidics chip channels, those with lower motility are progressively filtered out. Consequently, only sperm with optimal motility reached the final stage of the process. Furthermore, better sperm morphology was also observed after using the chips compared to SU/DG, which could also be attributed to the selection process, as they passed through the channels (with poor morphology sometimes being associated with impaired motility). Regarding SDF, the results favored the use of microfluidics chips, which aligns with previous studies [10,13]. This suggests that the damage caused by sample manipulation when following SU/DG protocols could be reduced with the use of microfluidics thanks to the diminished manipulation. However, a few other studies did not find significant differences [1,25], as suggested by the moderate risk of bias assessed in those studies.

In contrast, reproductive outcomes yielded more inconsistent results, which may be partly attributed to the substantial variation in the number of available publications and the total sample size across the outcomes. The findings showed an improved fertilization rate following the use of microfluidics, according to previous studies [4,40]. These findings suggest that the improvement in semen parameters, particularly the reduction in SDF, helps select higher-quality sperm for ICSI, thereby increasing the chances of successful fertilization. As for embryo euploidy, no significant differences were found, suggesting that the improvement in semen parameters did not impact the chromosomal content of the embryos. Nonetheless, some authors demonstrated a positive effect in their results [4,36].

Beyond this point, fewer significant differences were observed, which may be due to the limited number of studies that analyzed pregnancy, miscarriage and LBR. Improvements in the semen parameters after the use of microfluidics do not appear to significantly influence the biochemical pregnancy rate, which is consistent with the literature [24,45]. In contrast, microfluidics seems to have a positive impact on implantation [4], clinical pregnancy [1,36] and ongoing pregnancy [4]. However, some authors report contradictory findings [14,24,41,42]. Studies showing statistically significant effects for these outcomes tend to have wider 95% confidence intervals, whereas those with narrower intervals (indicating higher precision) do not report a beneficial effect of microfluidics. Therefore, these findings should be interpreted with caution.

Once implantation occurred, no significant differences were observed in the miscarriage rate, whether analyzed per cycle or per pregnancy. Therefore, the improvement achieved in the semen parameters does not appear to be crucial in influencing the results of this outcome, although one author did find a beneficial effect in the microfluidics group [45]. Finally, for LBR, similar rates were found per first ET and per concluded cycle in both groups (microfluids and SU/DG). In contrast to LBR per cycle and per all ET, where microfluids showed a beneficial effect, which has been supported by previous studies [16,35,36]. Partly, the current findings support the use of microfluidics, suggesting that the reduction in SDF and improvement in other semen parameters positively impact the LBR. However, discrepancies in the results demonstrate that no conclusive results can be drawn regarding this outcome, highlighting the need for further studies to analyze it, as the number of publications pertaining to the LBR was the lowest.

So far, the improvement in seminal parameters evidenced by microfluidics chip processing has not been fully reflected in reproductive outcomes. Overall, this meta-analysis offers a broader perspective on the findings from previous studies on the topic. It is essential to highlight that, when reviewing the results, factors such as the effect size, the number of publications available, sample size (N), dispersion in the 95%CI and data quality based on risk of bias should be considered, in addition to variations in the study populations or the use of different microfluidics chips (ZyMot, FertileChip, laboratory-made devices or other commercial brands). These differences among publications are limitations of the meta-analysis, as they may influence the conclusions drawn.

Based on the analysis conducted in this meta-analysis, the conclusions suggest that microfluidics may offer advantages in the seminal parameters, improving sperm capacitation. This, along with the expectation that reduced sample manipulation leads to less sperm DNA damage, could potentially enhance reproductive outcomes, but further studies are needed. It should also be noted that some chips seem to offer better results overall. It remains uncertain whether these improvements are significant enough to justify its routine clinical use, despite its clear advantage in freeing up technicians to focus on other tasks. Instead, it may be more appropriate to consider microfluidics chips in specific cases such as high SDF, poor semen quality and male factor infertility with a previous history of failed cycles.

## 5. Conclusions

In conclusion, microfluidics techniques for sperm capacitation appear to enhance the semen parameters, thereby improving the quality of samples that will subsequently be used for ICSI. While the analysis of specific reproductive outcomes suggests a beneficial effect, certain outcomes appear to remain unaffected, possibly due to the limited number of publications and the results of their quality assessment. Consequently, further research is necessary to clarify the effect of microfluidic devices on reproductive outcomes. From a practical standpoint, microfluidics can streamline semen preparation and reduce laboratory workloads, though this aspect has not been formally evaluated. Nonetheless, their use entails additional costs for clinics and patients. For this reason, rather than being integrated into routine clinical practice, their use should be considered in selected cases (such as patients with high SDF, poor sperm morphology or motility or previous cycle failure). Thus, while their use is promising and should be viewed favorably, it must be implemented with caution and subject to ongoing assessment.

## Figures and Tables

**Table 1:** Summary of the obtained results (CI 95%) after the statistical analysis for each outcome studied. The use of microfluidics is associated with a lower sperm concentration, higher progressive and total motility, improved sperm morphology, reduced sperm DNA fragmentation (SDF), increased fertilization rates, higher implantation rates, better clinical and ongoing pregnancy rates (per ET), lower miscarriage/pregnancy rates, live birth rate (LBR) per cycle and LBR/all ET. No significant influence has been detected on the euploidy rate, biochemical pregnancy rate, miscarriage rate per cycle, LBR after first transfer or LBR/concluded cycles. In bold letters are those estimates with statistically significant differences between groups.

Result	Units	Estimator	95% CI	Number of Articles (Sample Size)
**Seminal parameters**				
Concentration	M/mL	MD	**−15.95 [−19.28, −12.61]**	19 (1200)
Progressive motility	%A+B	MD	**14.50 [7.84, 21.71]**	8 (548)
Total motility	%	MD	**10.68 [6.04, 15.31]**	13 (832)
Morphology	%	MD	**1.41 [0.67, 2.16]**	7 (396)
SDF	%	MD	**−9.98 [−13.19, −6.76]**	15 (593)
**Reproductive outcomes after ICSI**				
Fertilization rate	%	OR	**1.22 [1.01, 1.46]**	12 (40,748)
Embryo euploidy rate	%	OR	1.34 [0.88, 2.04]	7 (13,813)
Biochemical pregnancy rate/ET	%	OR	1.23 [0.84, 1.80]	8 (1189)
Implantation rate	%	OR	**4.51 [1.42, 14.37]**	6 (400)
Clinical pregnancy rate/ET	%	OR	**1.73 [1.22, 2.45]**	16 (2333)
Ongoing pregnancy rate/ET	%	OR	**1.99 [1.03, 3.83]**	6 (973)
All types misscarriage rate/cycle	%	OR	0.84 [0.54, 1.31]	3 (758)
All types misscarriage rate/pregnancy	%	OR	0.71 [0.50, 1.02]	10 (800)
LBR/1st ET	%	OR	1.60 [0.80, 3.22]	2 (143)
LBR/1st cycle	%	OR	**1.59 [1.12, 2.24]**	2 (598)
LBR/all ET	%	OR	**1.65 [1.06, 2.55]**	3 (570)
LBR/concluded cycle	%	OR	1.03 [0.53, 2.00]	4 (244)

1st ET: first embryo transfer; authors only offered data from the first transferred embryo from each patient. All ET: all embryo transfer; authors offered data taking into count every embryo transfer from each patient, not necessarily being from the same cycle. 1st cycle: authors offered data for the outcome from patients who came into their clinic for their first cycle (they did not specify if there were any embryos left or not in the moment of publication of the results). Concluded cycle: authors offered data for this parameter from patients with concluded cycles (no embryos left from any of their cycles).

## Data Availability

Data are contained within the article.

## Appendix A

**Table A1:** Characteristics of the included studies in the meta-analysis for data extraction, assessing the comparation between seminal parameters and/or reproductive outcomes after sperm processing using microfluidic chips or conventional techniques (SU/DG).

Study	Design	Study Population Criteria: Inclusion (I)/Exclusion(E)	ART	Microfluidic Chip	Control	Measured Parameters
Anbari et al., 2021 [20]	Prospective randomized pilot study	I: couples with EOD E: severe male factor N = 95 couples	ICSI	Others	SU	Seminal parameters; reproductive outcomes
Asghar et al., 2014 [32]	Prospective non-randomized study	I: male volunteers E: severe male factor N = 12	-	Others	SU	Seminal parameters
Aydin et al., 2022 [34]	Prospective randomized study	I: IVF couples E: 3 or more IVF failures N = 128 couples	ICSI	Fertile Plus	SU	Reproductive outcomes
Dadkhah et al., 2023 [46]	Prospective non-randomized study	I: Fertility patients E: severe male factor N = 18	-	Others	SU + DG	Seminal parameters
Dev Nayar et al., 2021 [1]	Prospective randomized study	I: IVF couples E: severe male factor N = 300 couples	ICSI	Others	DG	Reproductive outcomes
Doostabadi et al., 2022 [21]	Prospective non-randomized study	I: males with DNA fragmentation index (DFI) ≥ 30% E: others N = 20 couples	-	Others	SU	Seminal parameters
Escudé-Logares et al., 2024 [44]	Retrospective study	I: Cycles with donated oocyte E: Depending on the analysis males under or more than 45 years old N = 132 cycles	ICSI	ZyMot	DG	Reproductive outcomes
Göde et al., 2019 [22]	Retrospective study	I: couples undergoing AI cycles E: IVF/ICSI couples N = 133 couples	AI	Fertile Plus	DG	Seminal parameters; reproductive outcomes
Göde et al., 2020 [23]	Prospective non-randomized study	I: Male volunteers E: severe male factor N = 201	-	Fertile Plus	SU + DG	Seminal parameters
Godiwala et al., 2022 [28]	Retrospective cohort study	I: couples with previous ICSI failures after DG E: others N = 176	ICSI	ZyMot	DG	Seminal parameters; reproductive outcomes
Godiwala et al., 2023 [29]	Prospective randomized sibling study	I: ICSI + PGT-A or PGT-M couples aged 18–42 years with ≥6 mature oocytes E: non PGT-A cycles N = 158 couples	ICSI	ZyMot	DG	Reproductive outcomes
Godiwala et al., 2024 [24]	Retrospective randomized sibling study	I: ICSI + PGT-A couples E: non PGT-A cycles N = 106 couples	ICSI	ZyMot	DG	Seminal parameters; reproductive outcomes
Keating et al., 2021 [4]	Retrospective study	I: ICSI couples E: donated oocytes/sperm N = 114 couples	ICSI	Others	DG	Seminal parameters; reproductive outcomes
Keskin et al., 2022 [35]	Retrospective observational study	I: patients with two or more ICSI failures + PGT-A and SDF > 30% E: rest N = 243 cycles	ICSI	Fertile Plus	DG	Reproductive outcomes
Kishi et al., 2015 [12]	Prospective non-randomized study	I: male volunteers E: - N = 10	-	Others	SU	Seminal parameters
Kocur et al., 2023 (1) [36]	Prospective non-randomized study	I: male volunteers undergoing sperm cryopreservation utilizing microfluidics E: - N = 2238 (98 microfluidics; 2140 DG)	-	Others	DG	Seminal parameters
Kocur et al., 2023 (2) [25]	Prospective non-randomized study	I: couples undergoing ICSI + PGT-A cycles, with >50% aneuploid embryos in previous cycles E: rest N = 57 couples	ICSI	ZyMot	DG	Seminal parameters; reproductive outcomes
Kothamasu et al., 2023 [8]	Retrospective study	I: couples undergoing ICSI cycles E: severe male factor N = 357 embryo transfers	ICSI	Others	DG	Reproductive outcomes
Lara-Cerrillo et al., 2023 [45]	Retrospective cohort study	I: couples undergoing ICSI with previous failures and >60% SDF E: rest N = 28 couples	ICSI	ZyMot	SU + DG	Reproductive outcomes
Meseguer et al., 2021 [30]	Prospective non-randomized pilot study	I: male volunteers aged 35 +/− 8 years E: - N = 25	-	Others	DG	Seminal parameters
Mirsanei et al., 2022 [10]	Prospective non-randomized study	I: depending on the analysis couples undergoing their 1st ICSI cycle/couples with previous ICSI failures E: PCOS, endometriosis, severe male factor N = depending on the analysis 10/15 couples, respectively	ICSI	Fertile Plus	DG	Seminal parameters; reproductive outcomes
Özaltin et al., 2023 [41]	Prospective randomized cohort study	I: couples undergoing ICSI cycles, women aged 18–43 years, no disorders, normal hormone panel, no severe male factor E: rest N = 213 couples	ICSI	Fertile Plus	SU + DG	Reproductive outcomes
Ozcan et al., 2021 [42]	Retrospective study	I: couples undergoing ICSI cycles with male factor infertility E: - N = 181 couples	ICSI	Fertile Plus	DG	Reproductive outcomes
Palmerola et al., 2020 [26]	Retrospective cohort study	I: ICSI + PGT-A couples E: non PGT-A cycles N = 167 cycles	IVF	Others	DG	Reproductive outcomes
Pardiñas et al., 2023 [13]	Prospective non-randomized study	I: ART patients E: - N = 27	-	ZyMot	SU	Seminal parameters
Parrella et al., 2019 [11]	Prospective non-randomized study	I: depending on the analysis couples undergoing ICSI + PGT-A cycles/ICSI cycles without PGT E: - N = 9 couples (depending on the analysis, 5 and 4, respectively)	ICSI	ZyMot	DG	Seminal parameters; Reproductive outcomes
Pavlovic et al., 2020 [37]	Retrospective cohort study	I: Couples undergoing ICSI with microfluidics, after DG E: - N = 749 MII oocytes	ICSI	Others	DG	Reproductive outcomes
Phairatana et al., 2023 [47]	Prospective non-randomized study	I: healthy men with normal semen profiles E: severe male factor N = 19 males	-	Others	DG	Seminal parameters
Pujol et al., 2022 [5]	Blinded split pilot study	I: patients with ≥ 60% dsSDF E: rest N = 9 males	-	ZyMot	SU	Seminal parameters
Quinn et al., 2018 [48]	Prospective non-randomized study	I: Infertile men from their medical center E: others N = 70 males	-	ZyMot	DG + SU	Seminal parameters
Quinn et al., 2022 [14]	Prospective randomized study	I: couples undergoing ICSI, inclusive with severe male factor E: donated oocytes/sperm N = 386 couples	ICSI	ZyMot	DG	Reproductive outcomes
Schiewe et al., 2021 [38]	Retrospective study	I: couples undergoing ICSI + PGT-A cycles E: - N = 3219 cycles	ICSI	ZyMot	DG	Reproductive outcomes
Sheibak et al., 2024 [27]	Prospective non-randomized study	I: 100 male patients (50 normozoospermic, 50 male factor infertility) E: - N = 100 samples	-	Others	SU/DG	Seminal parameters
Shirota et al., 2016 [31]	Prospective non-randomized study	I: healthy male volunteers E: - N = 37	-	Others	SU	Seminal parameters
Vasilescu et al., 2023 [39]	Prospective non-randomized study	I: male volunteers E: - N = 33	-	Others	SU	Seminal parameters
Yalcinkaya et al., 2019 [16]	Prospective sibling study	I: couples undergoing ICSI cycles, female patients <42 years old, with at least 5 MII oocytes E: severe male factor N = 81 patients	ICSI	Fertile Plus	SU	Reproductive outcomes
Yetkintel et al., 2019 [43]	Prospective randomized study	I: couples with EOD, women aged 20–40 years, patients with 1st or 2nd cycle E: rest N = 122 couples	ICSI	Fertile Plus	SU	Reproductive outcomes
Yildiz and Yuksel et al., 2019 [40]	Prospective randomized study	I: depending on the analysis patients undergoing their 1st ICSI cycle/patients with at least 2 previous cycle failures E: - N = 428 couples	ICSI	Fertile Plus	DG	Reproductive outcomes
Zaha et al., 2023 [33]	Retrospective study	I: patients undergoing ICSI cycles E: - N = 239 couples	ICSI	ZyMot	DG	Seminal parameters

ART: artificial insemination technique; ICSI: intracytoplasmatic microinjection; IVF: in vitro fertilization; AI: artificial insemination; SU: swim up; DG: density gradients; SDF: sperm DNA fragmentation; dsSDF: double-stranded sperm DNA fragmentation; PGT-M: preimplantation genetic testing for monogenic diseases; PGT.
